# New Curcumin Analogue (PAC) Inhibits *Candida albicans* Virulence, Restricts Its Adhesion Potential, and Relieves Oral Epithelial Cell Inflammation and Defense Mechanisms

**DOI:** 10.3390/antibiotics14050495

**Published:** 2025-05-12

**Authors:** Ghazoua Mezni, Hawraa Issa, Manal Dahdah, Anaïs Poulin, Adam Daïch, Abdulaziz Alamri, Mahmoud Rouabhia, Abdelhabib Semlali

**Affiliations:** 1 Groupe de Recherche en Écologie Buccale, Faculté de Médecine Dentaire, Université Laval, Québec, QC G1V0A6, Canada; ghazoua.mezni.1@ulaval.ca (G.M.); hawraa.issa.1@ulaval.ca (H.I.); manal.dahdah.1@ulaval.ca (M.D.); anais.poulin.1@ulaval.ca (A.P.); mahmoud.rouabhia@fmd.ulaval.ca (M.R.); 2 Normandie Univ., UNILEHAVRE, INC3M FR 3038 CNRS, URCOM, 76600 Le Havre, France. UR 3221, UFR ST, BP: 1123, 25 rue Philipe Lebon, 76063 Le Havre Cedex, France; adam.daich@univ-lehavre.fr; 3 Biochemistry Department, College of Science, King Saud University, P.O. Box 2455, Riyadh 11451, Saudi Arabia; abalamri@ksu.edu.sa

**Keywords:** PAC, *C. albicans*, virulence, SAPs genes, cytokines, anti-microbial β-defensins

## Abstract

**Objectives:** The oral cavity hosts one of the most complex microbial communities in the body. A disruption of the balance favors the growth of pathogenic species, contributing to oral diseases. The rise in microbial resistance has limited the effectiveness of conventional treatments, shifting the interest to natural product-based alternatives. Given its superior bioavailability and bioactivity in other models, this study investigates the antifungal potential of a novel curcumin derivative, PAC (3,5-bis(4-hydroxy-3-methoxybenzylidene)-*N*-methyl-4-piperidone), and studies its impact on host–pathogen dynamics and host defense mechanisms. **Methods:** *Candida albicans* was used as the model organism. Viability, growth kinetics, and colony formation were evaluated using optical density, agar culture, and MTT assay. Biofilm formation was assessed through electron microscopy and total sugar quantification. The morphological transition from hyphae to the less virulent blastospore was monitored using an optical microscope. The gene expression of adhesion factors and host defense markers was analyzed using RT-PCR. **Results:** PAC impairs *C. albicans* viability and reduces virulence by compromising biofilm formation and ensuring phenotypic transition to a blastospore form. Also, PAC controls *C. albicans* growth via necrosis/ROS pathways. As a result, PAC appears to repress host–pathogen interaction by downregulating SAPs, EAP1, and HWP1 adhesion genes, thus relieving the need to activate gingival epithelial cell defense mechanisms. This is highlighted by recording baseline levels of IL-6, IL-8, and IL-1β cytokines and antimicrobial β-defensin peptides in the presence of less virulent candida forms. **Conclusions:** PAC effectively reduces *C. albicans* virulence by limiting biofilm formation and adhesion while minimizing inflammatory responses. These findings support its potential as a promising therapeutic agent for infectious disease control.

## 1. Introduction

As the second largest and most diverse microbiota after the gastrointestinal tract, the oral cavity houses no less than 770 species, ranging from opportunists to commensals and pathogens [1]. In cases of dysbiosis, initially, non-pathogenic microorganisms are liable to transform into extremely virulent entities, capable of evading elimination by the immune system [2]. Various factors favor activating virulence characteristics and cause an imbalance toward unusual flora [3,4]. With greater precision, equilibrium or homeostasis can be influenced by host-related elements such as dry mouth, inadequate denture cleaning, and local (topical) steroid treatments [5]. In addition, failure in systemic defense mechanisms, resulting from the prolonged use of certain drugs or immunosuppressive conditions, can facilitate the establishment of infections [6,7,8]. Among a long list of microorganisms, *Candida albicans* (*C. albicans*) stands out as the most prevalent human pathogenic fungus, inducing a variety of diseases, spanning from simple superficial mucosal infections to life-threatening systemic infections [9]. Indeed, this opportunistic yeast species is found in 90% of oral cavity isolates and on the surface of various organs such as the skin, upper respiratory tract, intestines, and vagina [10,11,12]. Primary or localized oral candidiasis presents in several clinical forms. This includes acute types, chronic types, *candida*-associated lesions, and keratinized primary lesions superinfected with *candida* [13,14]. Chronic hyperplastic candidiasis is of particular concern because of its possible association with malignant transformation [15]. Briefly, chronic *candida* infection is thought to lead to epithelial dysplasia and, in some cases, progression to oral squamous cell carcinoma. This is possibly attributed to the production of carcinogenic substances such as nitrosamines and acetaldehyde [16].

*C. albicans* exhibits various elements of virulence, including its ability to antagonize the host immune response [17], the phenotypic transition from blastospore to hyphal morphology [18], the expression of adhesins [19], and the secretion of invasive molecules [20]. Secreted aspartyl proteases (SAPs), derived from a family of 10 members (SAP1-SAP10), are common virulence determinants. These hydrolytic enzymes are involved in both mucosal and systemic infections. They are associated with adhesion, tissue damage, invasion, and immune evasion [21,22,23]. Evidence suggests that SAP expression patterns, determined by cell type and environmental factors, contribute to phenotypic switching and virulence [24,25]. While the importance of SAP2 lies in the digestion of a broad spectrum of proteinic substrates, comprising the extracellular matrix [22], SAP1 to SAP6 and SAP9 and SAP10 are all involved in adhesion to host cells [26]. Other genes are also demonstrated to be implicated in virulence, mainly at the level of biofilm formation, resulting from *C. albicans* attachment and growth. Examples include epithelial adhesin protein 1 (EAP1) and hyphal wall protein 1 (HWP1), which present glycosylphosphatidylinositol anchoring motifs and are responsible for *C. albicans* adhesion to various surfaces, namely medical devices and epithelial cells [27,28]. Due to their crucial role in biofilm buildup, these genes can be identified as excellent therapeutic targets [27,29]. Importantly, it was reported that *C. albicans* makes a multifactorial contribution to cancer development and progression, including oral cancer, by combining mechanisms of immune evasion, toxicity, tissue disruption, and the modulation of cellular signaling pathways [30,31].

Although various drugs are available to treat *candida* infections, such as polyenes (amphotericin B/AMB or nystatin), azoles (voriconazole and posaconazole), and echinocandins (caspofungin, micafungin, and anidulafungin), the efficacy of many of these antifungal agents is compromised due to the emergence of microbial resistance [32]. This led to more investments in exploring the therapeutic potential of a variety of synthetic or natural substances to combat infections. Among the proposed candidates is Curcuma (*Curcuma longa* L.), a well-known Southeast Asian plant used both as a spice and a medicinal herb. Its main active component is curcumin, which confers a variety of therapeutic properties. Curcumin, the yellow spice in turmeric, has demonstrated a wide spectrum of biological and pharmacological activities, such as antioxidant [33,34], anti-inflammatory [35], antimicrobial [36,37,38,39], and anticancer [40] ones, to treat neurodegenerative diseases, including Alzheimer’s [41], Parkinson’s [42], and cancers [33,34]. The therapeutic benefits of curcumin for the latter appear to be multifactorial due to the regulation of transcription factors, cytokines, and enzymes associated with nuclear factor-kappa B (NF-kB) activity. The potential problems hindering the clinical use of curcumin are its color, lack of water solubility, relatively low in vivo bioavailability, and poor absorption characteristics [43]. Because of the multiple therapeutic activities attributed to it, there is an active search for a “super curcumin” alternative, as curcumin remains an ideal lead compound for the design of more effective analogs. Several of them have been synthesized and evaluated for their application in multifunctional pharmacology for a variety of conditions, such as liver fibrosis, inflammation, cardiovascular diseases, and cancer [35,37], among which EF24 and PAC are excellent representatives. Both have enhanced bioavailability over curcumin and show more potent bioactivity, including anticancer and anti-inflammatory effects [39]. Other curcuminoid pyrazoles have been synthesized and described as new therapeutic agents in inflammatory bowel diseases targeting matrix metalloproteinases [38]. However, some roles of EF24 and PAC remain unclear, such as whether they induce or suppress reactive oxygen species (ROS) production and whether they stimulate or inhibit the mitogen-activated protein kinase (MAPK) pathway [36].

Recently, our research team revealed PAC’s therapeutic potential for managing oral cancer, either as an alternative treatment [44] or as a complement to chemotherapy [45]. While the link between this analog and cancer has been well established, its antifungal effect is unclear. In light of these observations, the present study aimed to investigate PAC’s effect on the growth and virulence of *C. albicans* and, consequently, the pathogen interaction with the oral mucosa and the activation of epithelial defense mechanisms.

## 2. Results

### 2.1. PAC Impairs Viability Together with Restricting Candida Growth and Colony Formation

**Figure 1:**
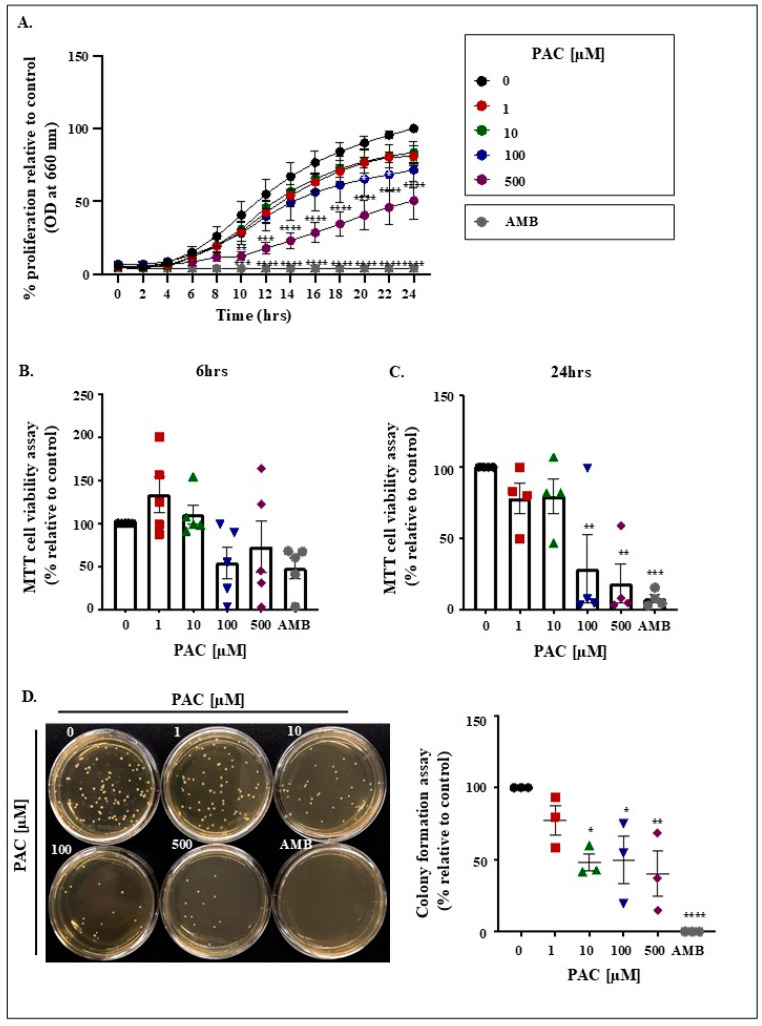
Effect of (PAC) on *C. albicans* growth and viability. (**A**). Optical density was recorded at 660 nm (n = 6) for 24 h to reflect the growth of *C. albicans*. (**B**). MTT cell viability and proliferation test (n = 4) performed after 6 h of treatment with different concentrations of PAC (1, 10, 100, and 500 µM). Results were confirmed by evaluation of (**C**). Colony-forming potential (n = 5). (**D**). Quantification of colony numbers. Results were reported as a percentage (100% represents the number of colonies in untreated cells at zero h) (n = 5). * *p* < 0.05, ** *p* < 0.005, *** *p* < 0.0005 and **** *p* < 0.00005 (compared with untreated *C. albicans* at the same time).

PAC inhibits the growth of *C. albicans* in a dose-dependent manner. This effect was evident as early as 10 h in the presence of PAC at 500 µM. Following 24 h, AMB scored 96% growth inhibition, while 100 and 500 µM PAC, respectively, ensured 29% and 50% proliferation suppression (Figure 1A). This observation is reinforced by the results of the MTT assay, where a significant reduction in viability, comparable to that of the positive control AMB, was revealed after incubation of the cells with 100 and 500 µM PAC for 24 h. At this level, the registered repression values were 71%, 82%, and 92% for the corresponding order of treatments: 100 µM PAC, 500 µM PAC, and AMB (Figure 1C). A general tendency toward cell loss was observed 6 h after drug exposure in reference to the untreated controls (Figure 1B). Crystal violet data, collected after 6 h incubation time, also revealed a significant reduction in *C. albicans* viability, ranging from 54% and 58% in 100 µM PAC and 500 µM PAC groups to 85% in the AMB group (Figure S1). The antifungal effect was further validated by the decrease in the number of colonies as evaluated on solid agar following the supplementation of increasing concentrations of the product in question for 24 h. Again, there was no significant difference between the highest concentration of PAC and AMB (Figure 1D).

### 2.2. PAC Compromises Biofilm Formation and Modifies Matrix Composition

**Figure 2:**
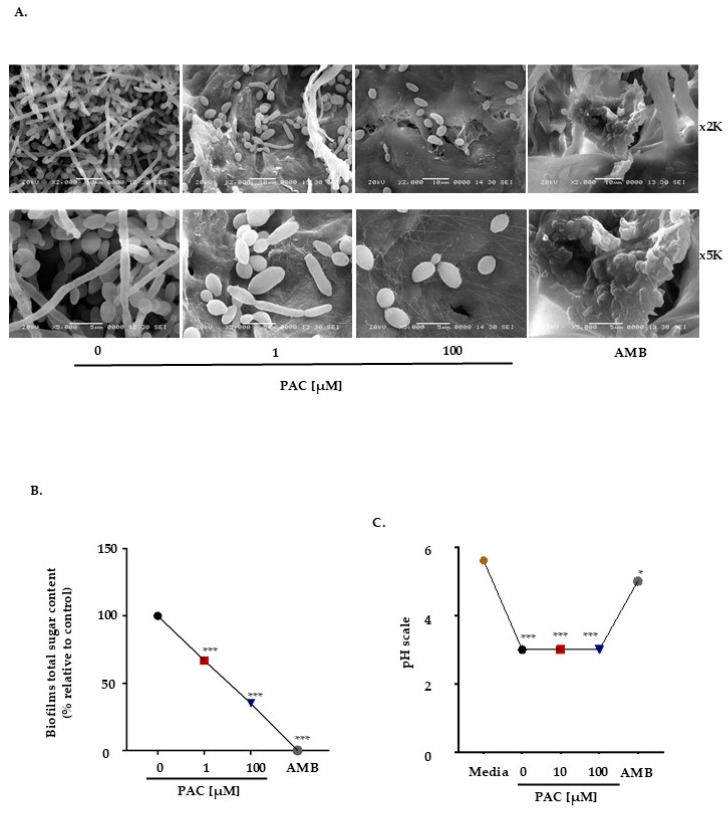
Effect of PAC on biofilm formation, acid production, and carbohydrate content. (**A**). Scanning electron microscopy analysis (n = 3) corresponding to 6 days of evolution of *candida* biofilms on porous membranes in the presence or absence of PAC and AMB. (**B**). Total sugar content of biofilms (n = 3). The antifungal agent AMB was used as a positive control, and comparisons are shown against untreated controls. (**C**) Evaluation of the pH variation in the medium under the effect of Viroelixir and AMB (n = 3). The antifungal agent AMB was used as a positive control, and comparisons are shown against untreated controls. All data are expressed as mean values ± SEM. * *p* < 0.05 and *** *p* < 0.001 are considered statistically significant.

Because PAC reduces *C. albicans* growth, it was essential to test its potential as an inhibitor of biofilm formation, being of primary relevance to pathogenicity. This was made possible by cultivating *C. albicans* for 6 days on collatape membranes, whether in the presence or absence of treatment. The scanning electron microscopy results showed that PAC significantly inhibits biofilm formation in a dose-dependent manner compared with untreated conditions. Notably, even at the lowest concentration tested, *candida* biofilms were significantly affected. At high concentrations, the *C. albicans* phenotype is more likely to be less virulent (blastospore). These data underline the efficacy of our compound and suggest its effectiveness as an antifungal agent with minimal adverse effects. The AMB antimycotic agent was adopted again as the positive control (Figure 2A). The next step was to investigate the effect of PAC on biofilm matrix composition, more specifically on polysaccharides, among others (proteins, lipids, membrane vesicles). Briefly, the total sugar content was measured using the colorimetric method of Dubois et al. after 6 days of treatment with different concentrations of PAC. Figure 2B shows that the biofilms’ total sugar content also decreases in a dose-dependent manner. As virulence factors have been shown to be influenced by pH variations, the correlation between these two parameters could be essential for a better understanding of PAC action. Although the decrease in medium pH, from 5.6 to 3 after 24 h of culture with *candida*, is probably due to the secretion of acidic substances, the addition of AMB almost restored the original Sabouraud medium pH. While the transition to a less acidic environment might directly impact pathogen elimination, its impact on virulence should not be ignored (Figure 2C).

### 2.3. PAC Ensures C. albicans Conversion to a Less Virulent Phenotype

**Figure 3:**
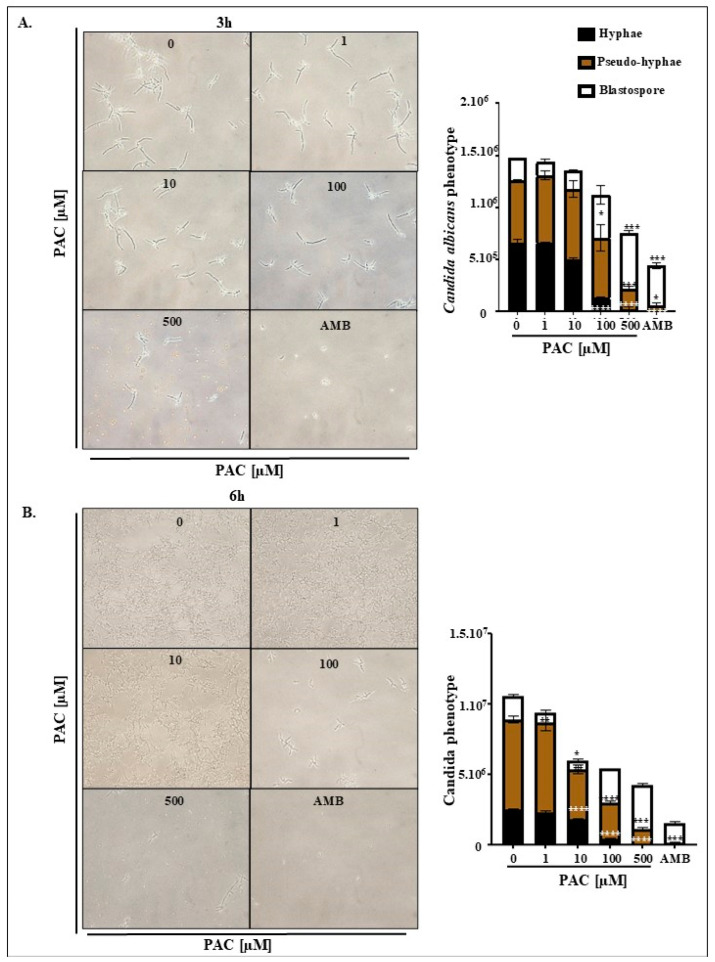
Effect of PAC on *C. albicans* morphology. (**A**) Morphological forms of C. *albicans* (blastospores, pseudohyphae, and hyphae) were detected microscopically and quantified (n = 3) after 3 h of exposure to PAC at concentrations of 1, 10, 100, and 500 µM under hyphal growth conditions. (**B**) Morphological forms of C. *albicans* (blastospores, pseudohyphae, and hyphae) were detected microscopically and quantified (n = 3) after 6 h of exposure to PAC under the same conditions. The antifungal agent amphotericin B (AMB) served as a positive control, and comparisons were made against untreated controls. Data are presented as mean values ± SEM. Statistical significance is indicated as * *p* < 0.05, ** *p* < 0.01, *** *p* < 0.001, and **** *p* < 0.0001.

The impact of PAC on phenotypic conversion was investigated, as hyphae forms are well documented to drive biofilm formation. Interestingly, we found that treatment significantly decreased hyphal and pseudohyphal density under serum-rich conditions compared to control groups in favor of a less virulent blastospore form. The effect was clearly shown at 3 h, when blastopores number doubled when exposed to 100 µM PAC. As for the 500 µM PAC and AMB treatments, the elevation corresponded to 257% and 187% (Figure 3A). We suppose that the smaller blastospore percentage detected in the presence of AMB might be due to the increased elimination of the *candida* pathogen. Furthermore, in both 500 µM PAC and AMB conditions, a negligible number of hyphae could persist. On a side note, hyphae size and ramifications were also reduced following PAC supplementation (Figure 3A,B).

### 2.4. PAC Alters Pathogen Adhesion Potential

**Figure 4:**
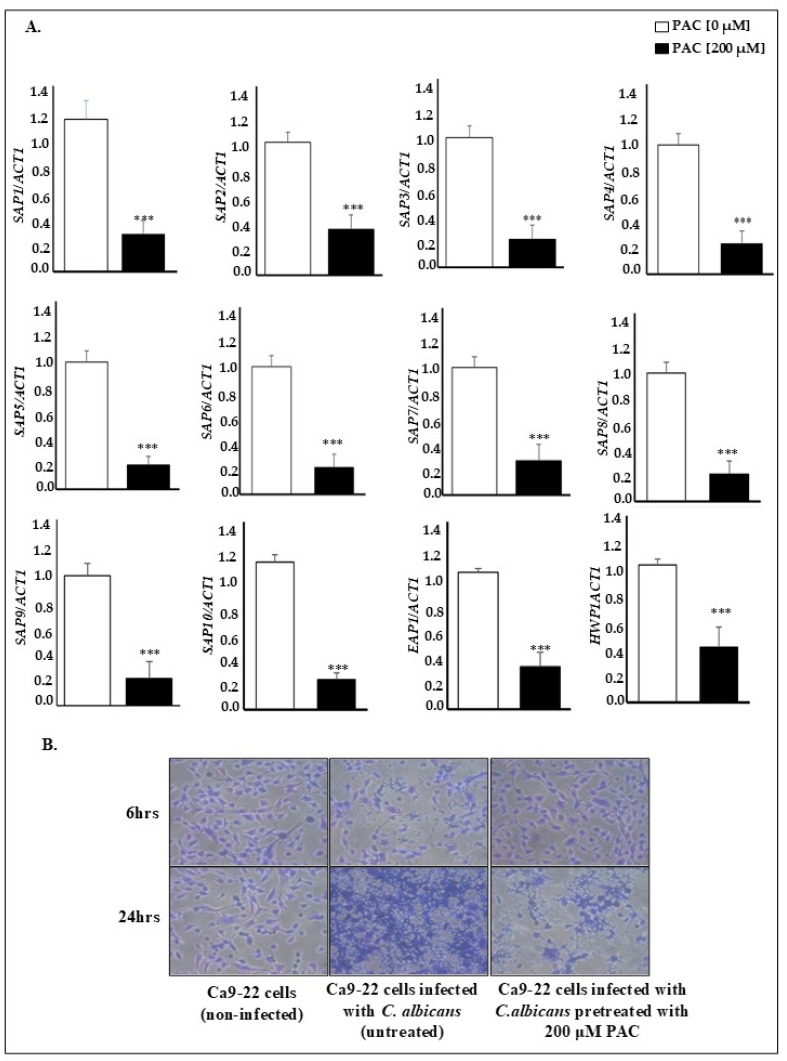
Effect of PAC on *C. albicans* gene expression and adhesion potential. (**A**) PAC decreased the expression of secreted aspartylproteinases (SAP, SAP1-SAP9), EAP1, and HWP1. *C. albicans* cells were treated with a concentration of 200 μM for 24 h at 37 °C, and then RNA was extracted and analyzed by qRT-PCR (n = 4). Gene expression was normalized to the housekeeping gene GAPDH and is presented as fold change relative to untreated *C. albicans* cells. *** *p* < 0.001 indicates statistically significant differences. (**B**) The adhesion potential of *C. albicans* to gingival epithelial cells was assessed microscopically (n = 3) using a crystal violet staining assay. Monolayers of gingival epithelial cells were exposed to *C. albicans* pretreated or not with PAC at a concentration of 200 μM for 6 and 24 h. Results represent mean ± SEM.

To investigate the mechanism by which PAC inhibits *C. albicans* virulence, a transcriptomic study of virulence genes was carried out using real-time PCR. Due to its contribution to biofilm formation and pathogenicity, adhesion was a critical event that warranted examination. Our data reported that PAC significantly reduces the expression of cell adhesion genes, mainly the secreted aspartyl protease (SAP) family members of hydrolytic enzymes (SAP1 to SAP 10) and EAP1 and HWP1, by at least 2 times. For instance, SAP1 expression decreased from 1.00 ± 0.14 in untreated controls to 0.288 ± 0.98 with PAC treatment, while that of SAP2 and SAP10 varied, respectively, from 0.98 ± 0.07 to 0.34 ± 0.1 and from 1.10 ± 0.05 to 0.22 ± 0.04. The expression of EAP1 and HWP1 genes decreased by 3.3- and 2.5-fold following 200 µM PAC treatment for 24 h (Figure 4A). This study was completed by assessing the impact of PAC on *C. albicans* attachment to an epithelial cell monolayer. In this respect, co-culture data revealed reduced adhesion to host cells under the action of PAC. This effect was noticeable at 6 h, where PAC hindering fungal agent attachment presented similarly to the condition unexposed to *candida*.

The observed mechanism could be attributable to the reduction in the expression of genes linked to cell adhesion (Figure 4B).

### 2.5. PAC Controls C. albicans Growth Through Necrotic and ROS Pathways

**Figure 5:**
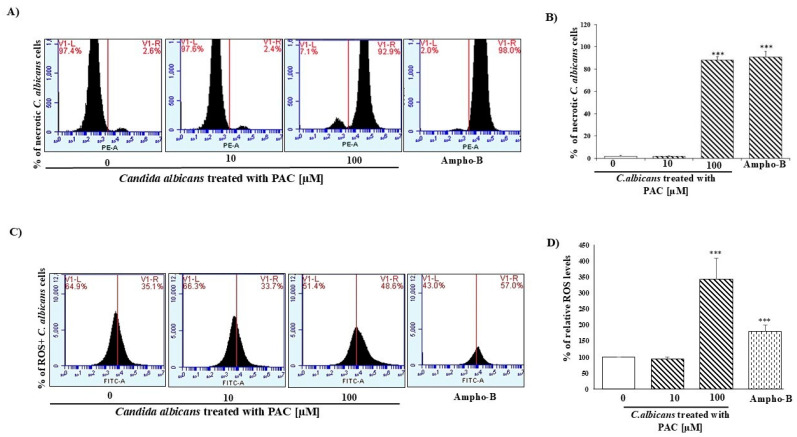
PAC controls *C. albicans* growth via necrosis/ROS pathways. (**A**) Flow cytometry analysis of Candida albicans viability and staining with propidium iodide (PI) after treatment with different concentrations of PAC and Ampho-B. The panels show viable and dead Candida albicans cells, marked or unmarked, under various treatment conditions (0, 10, 100 µM PAC, and Ampho-B). (**B**) Analysis data are presented as mean values ± SEM. Statistical significance is indicated as *** *p* < 0.0001. (**C**) Flow cytometry analysis of reactive oxygen species (ROS) production in *C. albicans* after treatment with different concentrations of PAC and Ampho-B. The panels show viable and dead Candida albicans cells, marked or unmarked, under various treatment conditions (0, 10, 100 µM PAC, and Ampho-B). (**D**) Summary of 4 independent experiments. Data were represented in percentages. (The untreated cells = 100%).

We investigated necrosis and oxidative stress in *C. albicans* after PAC treatment for 24 h. Based on our data in Figure 5, flow cytometry analyses showed (Figure 5A) a dose-dependent increase in the % of necrotic cells after PAC treatment, as the percentage of necrotic cells increased from 1.95 ± 0.45% in the untreated *C. albicans* culture to 88.0 ± 2.96% with 100 μM of PAC and 90.7 ± 5.16% with the positive control (Ampho-B). The ROS-positive cells increased following PAC treatment (Figure 5B). In addition, the percentage of ROS-positive cells was 100 ± 0.01% in the control cells and increased more than 4 times when *C. albicans* was treated with 100 μM of PAC to reach 425.96 ± 32.63% of ROS-positive cells, and 180.70 ± 18.98% when *C. albicans* culture was stimulated by the positive control (Ampho-B) (Figure 5B).

### 2.6. PAC Relieves Gingival Epithelial Cells’ Defense Mechanisms

**Figure 6:**
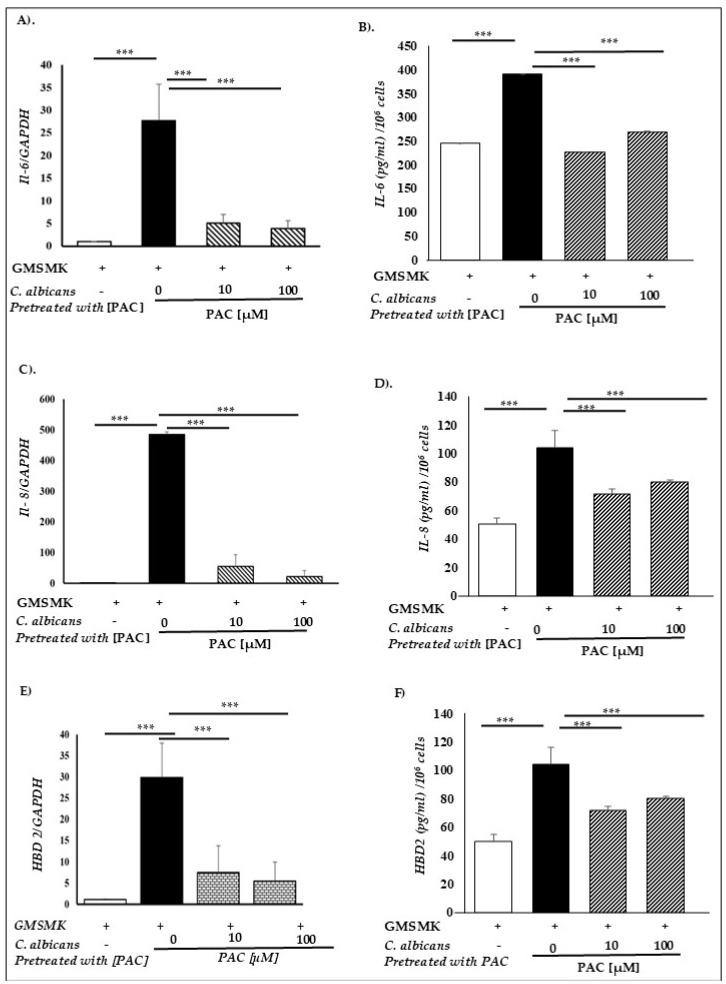
Effect of PAC on gene and protein expression of IL-1β, IL-6, IL-8, and HBD-2 in GMSMK cells after infection with *C. albicans*. (**A**) IL-6 gene expression at mRNA level in GMSMK cells after infection with *C. albicans* was analyzed by qRT-PCR and normalized to GAPDH. (**B**) IL-6 protein levels measured by ELISA. (**C**) IL-8 gene expression was analyzed by qRT-PCR and normalized to GAPDH. (**D**) IL-8 protein levels measured by ELISA. (**E**) Gene expression of HBD-2 was analyzed by qRT-PCR and normalized to GAPDH. (**F**) HBD-2 protein levels measured by ELISA. Experimental conditions include uninfected GMSMK cells (control), untreated *C. albicans*-infected GMSMK cells, and *C. albicans*-infected GMSMK cells pretreated with 10 µM and 100 µM PAC. For IL-6 and IL-8, gene expression levels decreased dose-dependent with increasing PAC concentration. However, protein levels for IL-6 and IL-8 were reduced with PAC 10 µM compared to untreated *C. albicans* but increased with PAC 100 µM. Despite this increase at 100 µM, protein levels remained significantly lower than in cells infected with untreated *C. albicans*. For HBD-2, gene and protein expression decreased significantly at 100 µM PAC compared with untreated *C. albicans*. Data are presented as mean ± SEM. *** *p* < 0.001.

Following infection, the gingival epithelial cells’ defense mechanisms can be resumed by their antimicrobial and pro-inflammatory potential. This is attributable to their capacity to secrete antimicrobial β-defensin peptides (hBD1, hBD2, hBD3, and hBD4) and pro-inflammatory cytokines (IL-1β, IL-6, and IL-8). Among multiple players, we chose to examine the effect of PAC exposure on the expression and secretion of IL-6, IL-8, and hBD2. To do so, the non-oncogenic human gingival epithelial cell line (GMSM-K) was used, real-time PCR was performed to quantify mRNA expression levels, and Elisa tests were carried out to confirm protein secretion levels. As shown in Figure 6, while wild-type violent *candida* cells were capable of significantly inducing IL-6, IL-8, and hBD2 expression and secretion by gingival epithelial cells, pre-exposure to PAC significantly blocked the activation of the aforementioned defense mechanisms. Although this might be the direct result of limiting the adhesion potential, it is undeniable that *C. albicans* pretreatment with 10 and 100 µM PAC for 24 h renders the pathogen less virulent. Likewise, *C. albicans* pretreated with PAC could not increase IL-1β, hBD1, hBD3, and hBD4 mRNA expression, similar to untreated *C. albicans* (Figures S2 and S3).

## 3. Discussion

The curcumin analog PAC was first developed by Youssef et al. in 2004 [46]. The initial study characterizing PAC was published in 2011 and focused on its effects against breast cancer [47]. Since then, PAC has demonstrated broad mechanisms of action and has shown efficacy across multiple cancer types [48,49], including oral cancer [44,45]. Notably, PAC has been reported to be five times more effective than curcumin in inducing apoptosis [47] while exhibiting selective cytotoxicity toward cancer cells [44]. These findings raise the possibility that PAC may also possess superior antifungal properties compared to curcumin. To the best of our knowledge, there is currently no evidence of the effects of PAC against infectious agents, including *C. albicans*. Beyond its proven functionality, the advantages of PAC lie in its enhanced chemical structure. Specifically, PAC replaces curcumin’s methylene and two carbonyl groups with an *N*-methyl-4-piperidone moiety, which contributes to increased stability and hydrophilicity. These modifications result in improved pharmacokinetic properties, including enhanced absorption, systemic bioavailability, and biodistribution. As reported by Al-Hujaily et al., PAC is 27 times more water-soluble than curcumin and exhibits superior absorption and retention in the bloodstream of mouse models. Notably, 60 min post-injection, PAC was found to be five times more stable than curcumin in circulation. Furthermore, PAC showed significant tissue uptake, stability, and retention in vital organs such as the liver, kidneys, and heart [47]. PAC’s structural and functional advantages are further highlighted by comparison with other curcumin analogs. For instance, EF24’s oral bioavailability is significantly limited by its lipophilic nature, poor water solubility, rapid degradation in biological environments (likely due to metabolism in the gastrointestinal tract and liver), and the action of efflux transporters that expel EF24 from intestinal cells [50]. Although novel drug delivery systems and EF24 derivatives have been developed to overcome these limitations [51], challenges with bioavailability remain. Based on the above, this study is the first to explore PAC’s potential antifungal activity against *C. albicans* and investigate its effects on key virulence factors, host–pathogen interactions, and key host defense mechanisms.

The first set of experiments clearly showed that PAC remarkably reduces the viability of *C. albicans* and limits its growth and virulence dose-dependently. Virulence was primarily represented by *candida*’s capacity to transition into a more virulent form known as hyphae. At this level, PAC was shown to decrease both hyphae density and length and ensure phenotypic conversion into a less virulent blastospore form. Of note, the morphological transition from a spheroid cell shape to hyphae has been documented to be responsible for biofilm formation and tissue invasion, therefore developing significant health problems [52]. Various natural products, notably from food, have shown their ability to target *C. albicans* virulence factors by inhibiting their hyphal forms. These natural products have been the subject of research, which opens promising prospects for preventing and treating *C. albicans* infections [53]. Considering these facts, PAC’s potential to limit biofilm formation was then confirmed, and its effect on the adhesion stage, which constitutes a primordial step in the formation of biofilms, was investigated. Indeed, recent studies have shown that oral pathogens exploit a diversity of factors involved in different stages of pathogenicity, including adhesins and the secretion system [54,55,56,57]. Mechanistically, we used a transcriptional approach to assess genes involved in pathogen adhesion. Overall, PAC was approved to downregulate aspartyl proteases SAP family genes (SAP1 to SAP10), considered as important virulence factors during mucosal infections, due to their implication in early biofilm formation events [58] and contribution to tissue damage [59]. Similar results were reported with curcumin, the source molecule of PAC, where modulatory effects on the main virulence factors associated with *C. albicans* pathogenicity were documented, more specifically, the inhibition of biofilm formation and the modulation of proteinase and phospholipase secretion [60,61]. For instance, reports have shown that HWP1, a key hyphal wall-associated element, was downregulated by curcumin [62]. PAC also decreased HWP1 and EAP1 mRNA expression levels, thus suggesting an alteration of *C. albicans* cell adhesion and tight binding to host cells [27,29]. Our results showed that PAC inhibits *C. albicans* adhesion to oral epithelial cells at this level. A similar study conducted by Shahzad et al. reported that curcumin possesses a strong ability to modify the adhesion of major periodontal pathogens and influence overall biofilm formation [62]. This current study is the first to show the link between curcumin analogs and *C. albicans* death through necrotic and ROS pathways, suggesting that necrosis and oxidative stress are the selected pathways of antifungal molecules to control fungal infections. By disrupting the biosynthesis of fungal cell wall components and preventing adhesion, PAC targeting the initial stage of the infection process is considered a promising strategy for preventing *C. albicans* infection.

In the current study, PAC influenced *candida* interaction with gingival epithelial cells, which argues in favor of its role in pathogen adhesion and host-mediated defenses. During oral infection, epithelial defenses are manifested by the release of pro-inflammatory cytokines and antimicrobial peptides, particularly those of the β-defensin family (hBDs). Infection persistence disrupts balance and plays a key role in the pathogenesis of many inflammatory diseases [63]. For this reason, the expression of the pro-inflammatory cytokines IL-1β, IL-6, and IL-8 and hBDs was analyzed in oral epithelial cells following infection with *C. albicans*. While *candida* infection increased pro-inflammatory cytokines and hBDs expression, less virulent forms, pretreated by PAC, were demonstrated to relieve the defense system. This presents similarities to *C. albicans’* response under monolaurin treatment [64]. Of note, antimicrobial peptides such as hBD-2 and hBD-3 have been demonstrated to reduce the invasive capacity of *C. albicans* and the modulation of host defense pathways [65]. hBDs are important for the control of early mucosal infection and play a critical role in the induction of innate inflammatory mediators [66,67,68,69]. A direct disruption of membrane integrity and permeabilization appears to be the predominant mechanism of β-defensins against microbes [70]. In this context, a groundbreaking study by Vylkova et al. showed that hBDs are capable of eliminating *C. albicans* in an energy-dependent and salt-sensitive manner without causing membrane disruption [71]. Hyphal formation and the presence of SAPs were shown to be important for the early induction of hBDs in oral epithelium [72]. In line with our results, curcumin’s anti-inflammatory properties have been well established in various cell types [73,74]. The natural product blocked IL-1 release in bone marrow stromal cells, colon epithelial cells, and human articular chondrocytes [75]. In general, it is worth pointing out that cytokines promote inflammation through the activation of the innate immune response and the induction of type 2 cyclooxygenase. In addition, it was reported that these factors increase the expression of adhesion molecules, nitric oxide synthesis, and the release of other cytokines [76]. Curcumin suppressed inflammatory cytokines production via the regulation of molecular targets and transcription factors [77]. In vascular smooth muscle cells, curcumin reverses LPS-induced inflammation via TLR4 activation, ERK1/2 and p38 MAPK phosphorylation inhibition, NF-κB nuclear translocation prevention, and NADPH-mediated intracellular ROS repression [78].

Evidence highlights the critical role of TLR4-mediated signaling, mainly through NF-κB, in protecting epithelial cells from *C. albicans* infection [79,80]. Besides NF-kB, receptor stimulation may lead to AP-1 activation. In addition, virulent *C. albicans* (hyphal forms) can activate mitogen-activated protein kinase 1 (MAPK1) and FOS signaling pathways, allowing the detection of tissue invasion [81]. Specifically, *candida* hyphae can invade the tissue by penetrating the epithelial cells known to produce cytokines via activating the MAPK1 pathway. Host cells can also respond by producing β-defensins with direct anti-*candida* activity [70]. Consequently, inflammatory cytokines and antimicrobial peptides were reduced to baseline levels compared to the response produced by virulent *C. albicans*. At this level, untreated *C. albicans* (virulent) significantly activated the defense system through TLR4/NF-κB pathways. Notably, although TLR4 was able to identify both virulent and non-virulent strains of *C. albicans*, the response varied considerably based on pathogen recognition and TLR4 receptor activation. TLR4 also recognized heat-killed *candida* due to conserved mannans on their surface [82]. This underscores the significance of TLR4 in differentiating fungal virulence and regulating host defenses appropriately.

## 4. Materials and Methods

### 4.1. Candida Strain and Epithelial Cell Culture

*C. albicans* (ATCC, MYA-2876), from *candida*-associated candidiasis, was grown in Sabouraud dextrose broth (Becton Dickinson, cat # 238230, Hong Kong, China ) containing 5 g/L peptone, 5 g/L peptic digest of animal tissue, 2 g/L pancreatic digest, and 20 g/L dextrose. The Sabouraud liquid medium was supplemented with 0.1% glucose and was used at pH equaling 5.6. Cultures were maintained in an incubator at 37 °C in the presence of 5% CO_2_. For the human epithelial cell lines, Ca9-22 (RIKEN BioResource Research Center, CVCL_1102, Ibaraki, Japan) and GMSM-K (provided by Dr. Daniel Grenier, Laval University, Quebec City, QC, Canada) were used between passages 3 and 20. Ca9-22 were grown in RPMI 1640 (Gibco, Burlington, ON, Canada, cat#10-040-CV) supplemented with 5% fetal bovine serum (FBS) (Thermo Fisher, Burlington, ON, Canada, cat ≠ 10 099-141), 0.2% amphotericin B (AMB) (Sigma-Aldrich, Oakville, ON, Canada, cat# A2942) and 0.2% Penicillin/Streptomycin (P/S) (Sigma-Aldrich, cat ≠ P4333). GMSM-K cells were maintained in DMEM (Gibco, Cat#11320033) media supplemented with 10% FBS, 0.2% AMB, and 0.2% P/S. 3,5-Bis(4-hydroxy-3-methoxybenzylidene)-*N*-methyl-4-piperidone, known as PAC, was obtained from the laboratory of Dr. Ibrahim Al-Jammaz (Riyadh, Saudi Arabia). PAC stock solution was prepared in DMSO, and working solutions at concentrations of 1, 10, 100, 200, and 500 µM were obtained by diluting the stock in Sabouraud medium. 5 µg/mL of the antimycotic agent AMB was used as a positive control.

### 4.2. C. albicans Growth Assay

A total of 2 × 10^4^ *candida* cells were seeded per well in a 96-well plate while being treated with increasing concentrations of PAC (1, 10, 100, and 500 µM). Automatic optical density readings were taken at a wavelength of 660 nm every 2 h for 24 h. This methodical approach guarantees a quantitative and reproducible assessment of the influence of PAC on *C. albicans* growth [83]. Three technical replicates and five biological replicates were carried out.

### 4.3. MTT Viability and Proliferation Assay

Cultures of *C. albicans*, initially at a density of 10^5^ cells/mL, were incubated for 6 and 24 h in the presence of increasing concentrations of PAC. Cell viability and proliferation were then assessed by measuring mitochondrial activity using the 3-(4,5-dimethylthiazol-2-yl)-2,5-diphenyltetrazolium bromide (MTT) assay. For further details, MTT (Sigma-Aldrich, cat ≠ C501/CT02) was added to the Sabouraud medium containing the cells at a concentration of 0.5 mg/mL and incubated for 3 h at 37 °C in the dark. Subsequently, the formazan crystals formed were dissolved in isopropanol containing 0.04% hydrochloric acid, and absorbance was measured at 550 nm using a Bio-Rad reader, following the method previously described by [44]. The results are represented as percentages using the following formula: % of proliferation = (treated cells OD/untreated cells OD) × 100. Four technical replicates and four to five biological replicates were carried out.

### 4.4. Colony Formation

The aim was to evaluate the efficacy of PAC (1, 10, 100, and 500 µM) on fungal growth compared with the control group (unstimulated *candida*) and the positive AMB control. Briefly, *C. albicans* cultures containing 10^3^ cells/mL were prepared in Sabouraud liquid medium, and then 20 µL of each condition was plated onto 60 mm diameter agar plates. Samples were incubated at 37 °C for 24 h before counting the colonies formed. The results are represented as percentages, with 100% corresponding to the number of colonies in untreated cells [66]. Three biological replicates were carried out.

### 4.5. Biofilm Formation by Transmission Electron Microscopy

Our previous study [84] reported that *C. albicans* were seeded on a porous collagen scaffold to promote biofilm formation and maintain the biofilm structure. For this purpose, 5 mm × 5 mm samples of porous scaffold (Collatape, Zimmer Dental Inc., Carlsbad, CA, USA) were placed on a 24-well plate. The scaffolds were then rinsed twice with culture medium, seeded with *C. albicans* (10^5^ cells), and incubated for 30 min at 30 °C without shaking to allow for adherence. Fresh Sabouraud medium was added to each well in the presence or absence of various concentrations of PAC (1 and 100 μM). Two controls were included in this study: the negative control corresponded to untreated *C. albicans*, while the positive control was AMB (5 μg/mL). The *C. albicans*-seeded scaffolds were then incubated for 6 days at 30 °C. The medium, PAC, and AMB were refreshed every 48 h. Following the 6-day culture period, scanning electron microscopy assessed biofilm formation after fixation with 2% (*v*/*v*) paraformaldehyde. Three biological replicates were carried out.

### 4.6. Assessment of Morphological Transition

As described in our previous study [85], to assess the influence of PAC on the phenotypic transition from hyphae to blastospore, 10^5^ cells were cultured in 1 mL Sabouraud broth in the presence of different PAC concentrations (1 µM, 10 µM, 100 µM, and 500 µM). The medium was enriched with 10% FBS, which promotes virulence and hyphal induction. Control conditions were either unstimulated (without PAC) or treated with the antifungal agent AMB. Cultures were examined under the microscope and photographed after incubation at 37 °C for 3 and 6 h. Based on cell morphology, cells were classified as blastospores, pseudohyphae, and hyphae. Three biological replicates were carried out.

### 4.7. pH Measurement

The cells (10^6^) were exposed to different PAC concentrations. After 24 h incubation, the supernatant was collected, and pH was measured using pH indicator strips (MColorpHast, 1-09535-0001). Three technical replicates and five biological replicates were carried out.

### 4.8. Measurement of Biofilm Matrix Composition: Total Sugars

As previously described, following 6 days of formation of *C. albicans* biofilms on collatape scaffolds, sonication was performed, and colorimetric determination of total sugars was conducted using the method described by Dubois et al. First, a calibration curve covering concentrations from 0 to 4000 mg/L glucose was established. Next, 200 µL of sonication product was mixed with 100 µL of phenol 5% and 500 µL of H_2_SO_4_. The mixture was incubated at 30 °C for 20 min and then cooled under water to 20 °C. Absorbance correlated with yellow-red complex formation was measured at 485 nm, and concentrations were determined by reference to the standard [86]. Three technical replicates and four biological replicates were carried out.

### 4.9. Quantitative Real-Time RT-PCR

*Candida* samples were grown at a density of 10^7^ cells in the presence or absence of the median inhibitory concentration (IC_50_) of PAC, determined at 200 µM. After 24 h at 37 °C, cells were collected by centrifugation for 10 min at 12,000 RPM. Each pellet was suspended in 500 µL of RLT lysis buffer (Qiagen, Toronto, ON, Canada, cat ≠ 74104) in the presence of 0.2 mL glass beads with a diameter measuring between 0.425 and 0.6 mm (Sigma-Aldrich, cat ≠ 1001776233). According to the method described previously [87], the samples were then vortexed and placed at −80 °C for 30 min before being subjected to sonication (15 one-minute cycles of sonication followed by incubation on ice for the same period) using a Mini Bead beater (RPI, cat ≠112011). After cell lysis, total RNA was extracted using the RNeasy plus Mini extraction kit (Qiagen, cat ≠ 74104), as per the manufacturer’s instructions. The isolated RNA’s purity, concentration, and quality were all measured using a Thermo Fisher Scientific NanoDrop 8000. Total RNA, at a rate of 1 µg per sample, was converted to complementary DNA (cDNA) using the iScriptTM Reverse transcription Supermix for RT-qPCR in accordance with the manufacturer’s protocol (Bio-Rad, Mississauga, ON, Canada, cat#1708841). Virulence genes were evaluated by quantitative RT-PCR. PCR reactions were performed in a total volume of 20 µL. Each sample consisted of 5 µL of cDNA, added to a reaction mixture of 10 μL of SYBR Green iQ supermix (Bio-Rad, cat ≠ 1708841), 1 μL of each primer (Midland Certified Reagent Company, Midland, TX, USA) (Table 1 and Table 2), and 4 μL of RNase-free water (Qiagen, cat ≠ 129115). Each reaction was performed in triplicate in a Bio-Rad MyCycler thermal cycler. The actin gene (ACT1) and GAPDH, demonstrating stable expression levels, were selected as reference genes for this study. The results were analyzed using the 2^−ΔΔCt^ relative expression method (Livak). Five biological replicates were carried out.

### 4.10. Adhesion to Host Cells

To investigate the effect of PAC on *C. albicans* adhesion potential to host cells, 3 × 10^5^ Ca9-22 cells were seeded in 6-well plates for 24 h without antibiotic and antifungal agents before adding 2 × 10^6^ *C. albicans*, pretreated or not, to PAC IC_50_ for 24 h. *C. albicans’* adhesion to cells was evaluated after 6 h of co-culture. Overall, the cells were washed 3 times with PBS1× to remove non-adherent *C. albicans* before taking photographs under an epifluorescent light. Four biological replicates were carried out

### 4.11. Cell Viability Analysis by Propidium Iodide (PI) Staining

Cell death in *Candida albicans* was evaluated using the Propidium Iodide Apoptosis Kit (BioLegend, San Diego, CA, USA). Briefly, after 24 h of treatment with PAC (0 µM, 10 µM, 100 µM) or amphotericin B (positive control), *C. albicans* cells (1 × 10^7^ cells/mL) were centrifuged at 500× *g* for 5 min and washed with sterile 1 × PBS. The cell pellets were then resuspended in 100 μL of PBS, stained with 10 μL of PI, and incubated for 15 min at room temperature in the dark. After staining, 400 μL of assay buffer was added to each tube, and flow cytometry analysis was performed using the BD Accuri C6 Plus Flow Cytometer (BD Biosciences, San Jose, CA, USA). A total of 50,000 events per sample was collected and classified as necrotic cells (PI^+^). Two biological replicates were performed.

### 4.12. Reactive Oxygen Species (ROS) Analysis by Flow Cytometry

Oxidative stress in *C. albicans* was assessed by flow cytometry using a total ROS detection kit (ImmunoChemistry Technologies, Burlington, ON, Canada), according to the manufacturer’s instructions. Briefly, after 24 h of treatment with PAC (0 µM, 10 µM, and 100 µM) or amphotericin B (positive control), *C. albicans* cells (1 × 10^7^ cells/mL) were centrifuged at 500× *g* for 5 min and washed with sterile 1 × PBS. The cells were then labeled with 10 μL ROS detection reagent (previously reconstituted in DMSO and diluted in assay buffer) with 490 μL of assay buffer 1 × and incubated in the dark at 30 °C for 1 h. After staining, samples were immediately analyzed using a BD Accuri™ C6 Plus Flow Cytometer (BD Biosciences, San Jose, CA, USA) with excitation at 488 nm. The experiment was performed in four biological replicates.

### 4.13. ELISA Assay

A total of 3 × 10^5^ GMSMK cells seeded in 6-well plates were incubated with 2 × 10^5^ *C. albicans*, pretreated or not, with varying concentrations of PAC for 24 h. Following 24 h of co-culture, the supernatants were collected, and the following thermo fisher ELISA kits were used, according to the supplier’s recommendations, to ensure the quantification of pro-inflammatory cytokines and antimicrobial peptides: IL-6 (Cat#88-7068-88), IL-8 (Cat#88-8086-88), and hBD-2 (cat#EK-072-37). The quantity of protein was normalized by 10^6^ cells, and the experiments were carried out 4 times.

### 4.14. Statistical Analysis

All experiments were repeated at least three times, with experimental values presented as mean ± SEM. Differences between the controls and treatment conditions were determined using the one-way ANOVA or the two-way ANOVA tests. GraphPad PRISM 9.4.0 software was used for this purpose. PCR data were analyzed using the 2^−ΔΔCt^ relative expression method (Livak). Values * *p* < 0.05, ** *p* < 0.01, *** *p* < 0.001, **** *p* < 0.0001 were considered statistically significant.

## 5. Conclusions

PAC exhibits promising antifungal activity against *C. albicans*, effectively inhibiting pathogen growth and virulence while suppressing host–pathogen interactions through the downregulation of hydrolytic enzymes and hyphal cell wall proteins. These findings support the potential integration of PAC into infectious disease control strategies. Future research should focus on elucidating the underlying metabolic processes and molecular mechanisms, particularly the role of the TLR4/NF-κB signaling pathway in modulating host immune responses. Additionally, exploring PAC in combination with conventional antifungals may help identify synergistic effects and optimize therapeutic outcomes. Finally, in vivo validation using a murine model of oral candidiasis is essential to confirm the clinical relevance and safety of PAC-based therapies.

## Figures and Tables

**Table 1:** Primer sequences corresponding to SAP family (1–9) and EAP1.

Gene for Candida	Primer Sequence (5′-3′)	Amp Size (bp)
*HWP1*	Forward-GACCGTCTACCTGTGGGACAGT Reverse-GCTCAACTTATTGCTATCGCTTATTACA	67
*EAP1*	Forward-CTGCTCACTCAACTTCAATTGTCG Reverse -GAACACATCCACCTTCGGGA	51
*SAP1*	Forward-TTTCATCGCTCTTGCTATTGCTT Reverse -TGACATCAAAGTCTAAAGTGACAAAACC	86
*SAP2*	Forward-TCCTGATGTTAATGTTGATTGTCAAG Reverse -TGGATCATATGTCCCCTTTTGTT	82
*SAP3*	Forward-GGACCAGTAACATTTTTATGAGTTTTGAT Reverse -TGCTACTCCAACAACTTTCAACAAT	87
*SAP4*	Forward-AGATATTGAGCCCACAGAAATTCC Reverse -CAATTTAACTGCAACAGGTCCTCTT	81
*SAP5*	Forward-CATTGTGCAAAGTAACTGCAACAG Reverse -CAGAATTTCCCGTCGATGAGA	77
*SAP6*	Forward-CCTTTATGAGCACTAGTAGACCAAACG Reverse -TTACGCAAAAGGTAACTTGTATCAAGA	101
*SAP7*	Forward: 5′-GAAATGCAAAGAGTATTAGAGTTATTAC Reverse: GAATGATTTGGTTTACATCATCTTCAACTG	196
*SAP8*	Forward: TCCCTGAAGACATTGATAAAAGAGC-3′ Reverse: AGAATCAACCACCCATAAATCAGAA-5′	278
*SAP9*	Forward-ATTTACTCCACAGTTTATCACTGAAGGT Reverse -CCACAAGAACCACCCTCAGTT	86
*SAP10*	Forward -CCCGGTATCCAATAGAATCGAA Reverse-TCAGTGAATGTGACGAATTTGAAGA	78
*ACT1*	Forward-GCTGGTAGAGACTTGACCAACCA-3′ Reverse: GACAATTTCTCTTTCAGCACTAGTAGTGA	87

**Table 2:** Primer sequences were used for qRT-PCR to evaluate the expression of inflammatory cytokines and β-defensin genes by gingival epithelial cells.

GMSM-K Genes	Primer Sequence (5′-3′)	Size (bp)
IL-1β	Forward: CTGTCCTGCGTGTTGAAAGA Reverse: TTGGGTAATTTTTGGGATCTACA	69
IL-6	Forward: TCTCCACAAGCGCCTTCG Reverse: CTCAGGGCTGAGATGCCG	203
IL-8	Forward: TTGGCAGCCTTCCTGATT Reverse: AACTTCTCCACAACCCTCTG	248
hBD-1	Forward: GCCTCTCCCCAGTTCCTGAA Reverse: GCAGAGAGTAAACAGCAGAAGGTA	82
hBD-2	Forward: TGTGGTCTCCCTGGAACAAAAT Reverse: GTCGCACGTCTCTGATGAGG	105
hBD-3	Forward: CTTCTGTTTGCTTTGCTCTTCCT Reverse: CTGTTCCTCCTTTGGAAGGCA	138
hBD-4	Forward: CACTCTACCAACACGCACCTAG Reverse: CGCAACTGGAACCACACACT	133
GAPDH	Forward: GGTATCGTCGAAGGACTCATGAC Reverse: ATGCCAGTGAGCTTCCCGTTCAGC	180

## Data Availability

All data generated or analyzed during this study are included in this published article.

## Supplementary Materials

The following supporting information can be downloaded at https://www.mdpi.com/article/10.3390/antibiotics14050495/s1. (Figure S1: Effect of PAC on biofilm formation, Figure S2: Effect of PAC on IL-1β gene expression in GMSMK cells after infection with *C. albicans*. Figure S3: Effect of PAC on gene expression of HBD-1, HBD-3 and HBD-4 in GMSMK cells after infection with *C. albicans*).

## Abbreviations

PAC	3,5-Bis(4-hydroxy-3-methoxybenzylidene)-*N*-methyl-4-piperidone
*C. albicans*	*Candida albicans*
SAPs	Secreted aspartyl proteases
HWP1	Hyphal wall protein 1
EAP1	Epithelial adhesin protein 1
RT-PCR	Reverse transcription polymerase chain reaction
NF-κB	Nuclear factor kappa-light-chain-enhancer of activated B cells
p38	p38 mitogen-activated protein kinases
JNK	c-Jun N-terminal kinase
IL-6, IL-8, IL-1β	Interleukin-6, Interleukin-8, Interleukin-1 beta
hBD	Human beta-defensin
AMB	Amphotericin B
RPMI	Roswell Park Memorial Institute medium
DMSO	Dimethyl sulfoxide
FBS	Fetal bovine serum
MTT	3-(4,5-dimethylthiazol-2-yl)-2,5-diphenyltetrazolium bromide
SEM	Scanning electron microscopy
XTT	2,3-Bis(2-methoxy-4-nitro-5-sulfophenyl)-5-[carbonyl(phenylamino)]-2H-tetrazolium hydroxide
PBS	Phosphate-buffered saline
DMEM	Dulbecco’s Modified Eagle Medium
FBS	Fetal bovine serum
TLR4	Toll-like receptor 4
CD284	Cluster of differentiation 284 (TLR4 marker)
FITC	Fluorescein isothiocyanate
RNA	Acide ribonucléique
DNA	Acide désoxyribonucléique
cDNA	ADN complémentaire
PCR	Polymerase chain reaction
Ct	Cycle threshold
bp	Base pair
