# Supplementary material for: New Curcumin Analogue (PAC) Inhibits Candida albicans Virulence, Restricts Its Adhesion Potential, and Relieves Oral Epithelial Cell Inflammation and Defense Mechanisms
Source: Antibiotics (Basel). 2025 May 12;14(5):495. doi: 10.3390/antibiotics14050495 (PMC12108166; doi:10.3390/antibiotics14050495)
Supplement: Supplementary file 1 [file antibiotics-14-00495-s001.zip › antibiotics-3569095-supplementary.pdf]

Suplementary data

A).

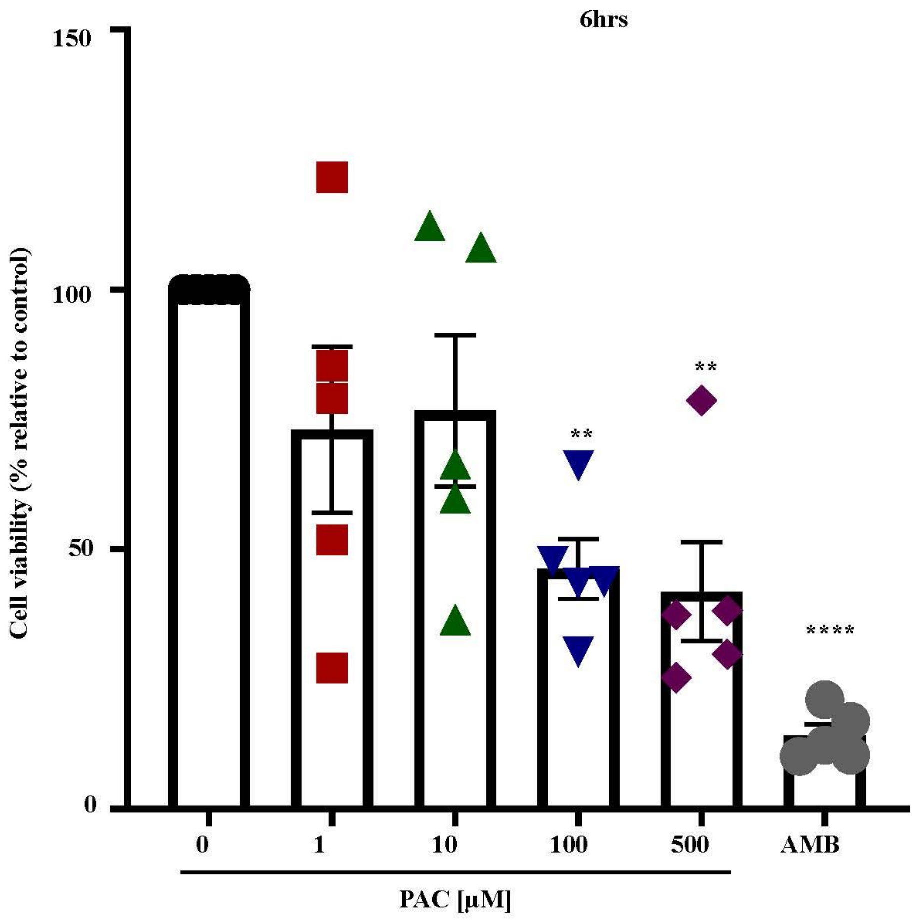

Supplementary Figure S1: Effect of PAC on biofilm formation. A). Crystal Violet staining (n=4) was performed after treatment of fungal cells with different concentrations of PAC (1, 10, 100, and 500 µM) for a total duration of 6 hours. The antifungal agent AMB served as positive control, and comparisons are shown against untreated controls. All data are expressed as mean values ± SEM. *p<0.05, **p<0.01 and ***p<0.001 are considered statistically significant.

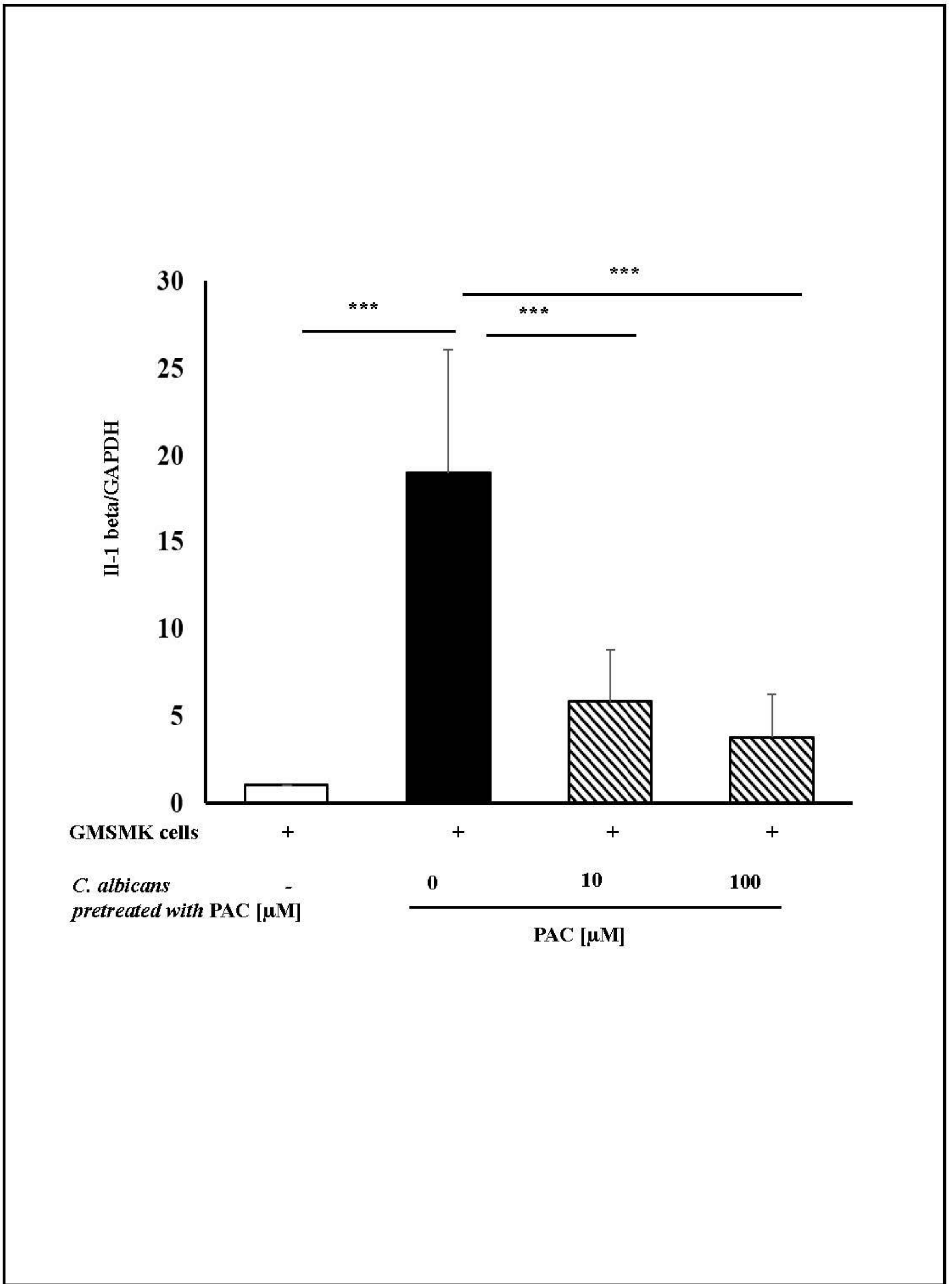

Supplementary Figure S2: Effect of PAC on IL-1β gene expression in GMSMK cells after infection with *C. albicans*. (A) IL-1β expression at mRNA levels measured by qRT-PCR. Data are presented as mean ± SEM. ***p < 0.001.

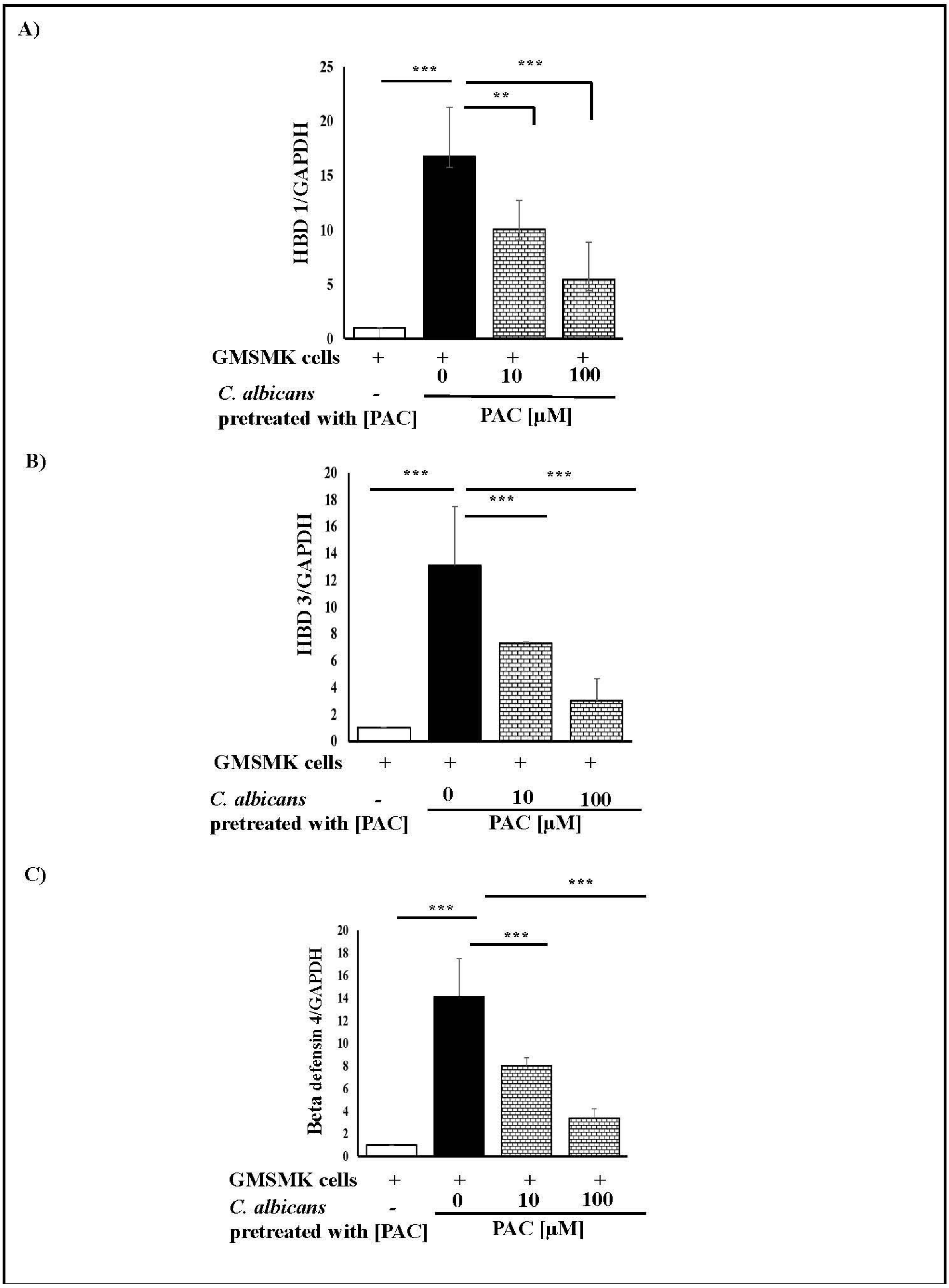

Supplementary Figure S3: Effect of PAC on gene expression of HBD-1, HBD-3 and HBD-4 in GMSMK cells after infection with C. *albicans*. (A) Gene expression of HBD-1 analyzed by qRT-PCR and normalized against GAPDH. (B) Gene expression of HBD-3 analyzed by qRT-PCR and normalized to GAPDH. (C) Gene expression of HBD-4 analyzed by qRT-PCR and normalized to GAPDH. Experimental conditions include uninfected GMSMK cells (control), untreated *C. albicans*-infected GMSMK cells and *C. albicans*-infected GMSMK cells pretreated with PAC at 10 μM and 100 μM. Gene expression levels of HBD-1, HBD-3, and HBD-4 decreased significantly with PAC treatment in a dose-dependent manner, particularly at 100 μM. Data are presented as mean ± SEM. ***$p < 0.001$.
